# Positive selection on the nonhomologous end-joining factor Cernunnos-XLF in the human lineage

**DOI:** 10.1186/1745-6150-1-15

**Published:** 2006-06-02

**Authors:** Adam Pavlicek, Jerzy Jurka

**Affiliations:** 1 Genetic Information Research Institute, Mountain View, CA 94043, USA

## Abstract

**Background:**

Cernunnos-XLF is a nonhomologous end-joining factor that is mutated in patients with a rare immunodeficiency with microcephaly. Several other microcephaly-associated genes such as *ASPM *and *microcephalin *experienced recent adaptive evolution apparently linked to brain size expansion in humans. In this study we investigated whether *Cernunnos-XLF *experienced similar positive selection during human evolution.

**Results:**

We obtained or reconstructed full-length coding sequences of chimpanzee, rhesus macaque, canine, and bovine *Cernunnos-XLF *orthologs from sequence databases and sequence trace archives. Comparison of coding sequences revealed an excess of nonsynonymous substitutions consistent with positive selection on *Cernunnos-XLF *in the human lineage. The hotspots of adaptive evolution are concentrated around a specific structural domain, whose analogue in the structurally similar XRCC4 protein is involved in binding of another nonhomologous end-joining factor, DNA ligase IV.

**Conclusion:**

*Cernunnos-XLF *is a microcephaly-associated locus newly identified to be under adaptive evolution in humans, and possibly played a role in human brain expansion. We speculate that Cernunnos-XLF may have contributed to the increased number of brain cells in humans by efficient double strand break repair, which helps to prevent frequent apoptosis of neuronal progenitors and aids mitotic cell cycle progression.

**Reviewers:**

This article was reviewed by Chris Ponting and Richard Emes (nominated by Chris Ponting), Kateryna Makova, Gáspár Jékely and Eugene V. Koonin.

## Open peer review

Reviewed by Chris Ponting and Richard Emes (nominated by Chris Ponting), Kateryna Makova, Gáspár Jékely and Eugene V. Koonin. For the full reviews, please go to the Reviewers' comments section.

## Background

Double-strand breaks (DSBs) are highly cytotoxic DNA lesions caused by ionizing radiation, spontaneous chromosomal breaks, activity of cellular endonucleases, or during replication of other DNA lesions such as single-strand breaks. If unrepaired, DSBs efficiently trigger arrest of cell cycle progression and cell death by apoptosis [1]. In response to this danger, cells have developed mechanisms that repair DSBs. In eukaryotic cells, there are two major groups of DSB repair pathways [2]: homologous recombination (HR) and nonhomologous end-joining (NHEJ). In contrast to HR, NHEJ does not require a highly identical undamaged partner DNA strand to repair DSBs and, after some processing, can ligate virtually any two DNA ends. This makes NHEJ a very efficient, yet error-prone DSB repair mechanism.

The lack of mutation in known NHEJ components in a patient with characteristic phenotypic effects of defective NHEJ lead to the conclusion that there must be at least one undiscovered component of the NHEJ pathway [3]. The search for this additional element lead to the recent discovery of a new NHEJ factor called Cernunnos-XLF [4,5]. Homozygous *Cernunnos-XLF *mutations are manifested by autosomal recessive immunodeficiency associated with mental retardation and microcephaly [4]. This 2q35 gene encodes a protein that interacts with the core NHEJ ligation complex composed of DNA ligase IV and XRCC4 [5,6]. The Cernunnos-XLF protein shows similarity to XRCC4 [5] and is homologous to the Nej1 NHEJ factor from yeast [6]. The locus seems to be present in all animals, most fungi, but not in plants.

The presence of microcephaly in patients prompted us to look closely for evolution of *Cernunnos-XLF *in primates, because several other genes linked to microcephaly-related disorders and brain size are under positive selection in hominoid primates and humans [7-15]. By comparing *Cernunnos-XLF *genes in five different mammalian species, we discovered strong evidence for adaptive evolution of this locus in the human lineage. Therefore, Cernunnos-XLF can be considered as yet another strongly selected factor, potentially contributing to increased skull and brain size in humans.

## Results and discussion

### Conservation of *Cernunnos-XLF *in mammals

Human (CAI99410), cow (XP_586059), and dog Cernunnos-XLF (XP_848099) proteins and the corresponding coding sequences (CDS) were extracted from Genbank. The macaque and chimpanzee copies were assembled from the Genbank trace archive and genome assembly, respectively (see Methods). Except for dog, all the genes appear to encode 299 aa long proteins; the predicted dog coding sequence contains an additional domain at the 5' end. Since this domain is not conserved in other species, it very likely represents an error in automated gene annotation and we shortened the dog ortholog to the 299 aa segment that is homologous to the remaining mammalian proteins.

**Figure 1:**
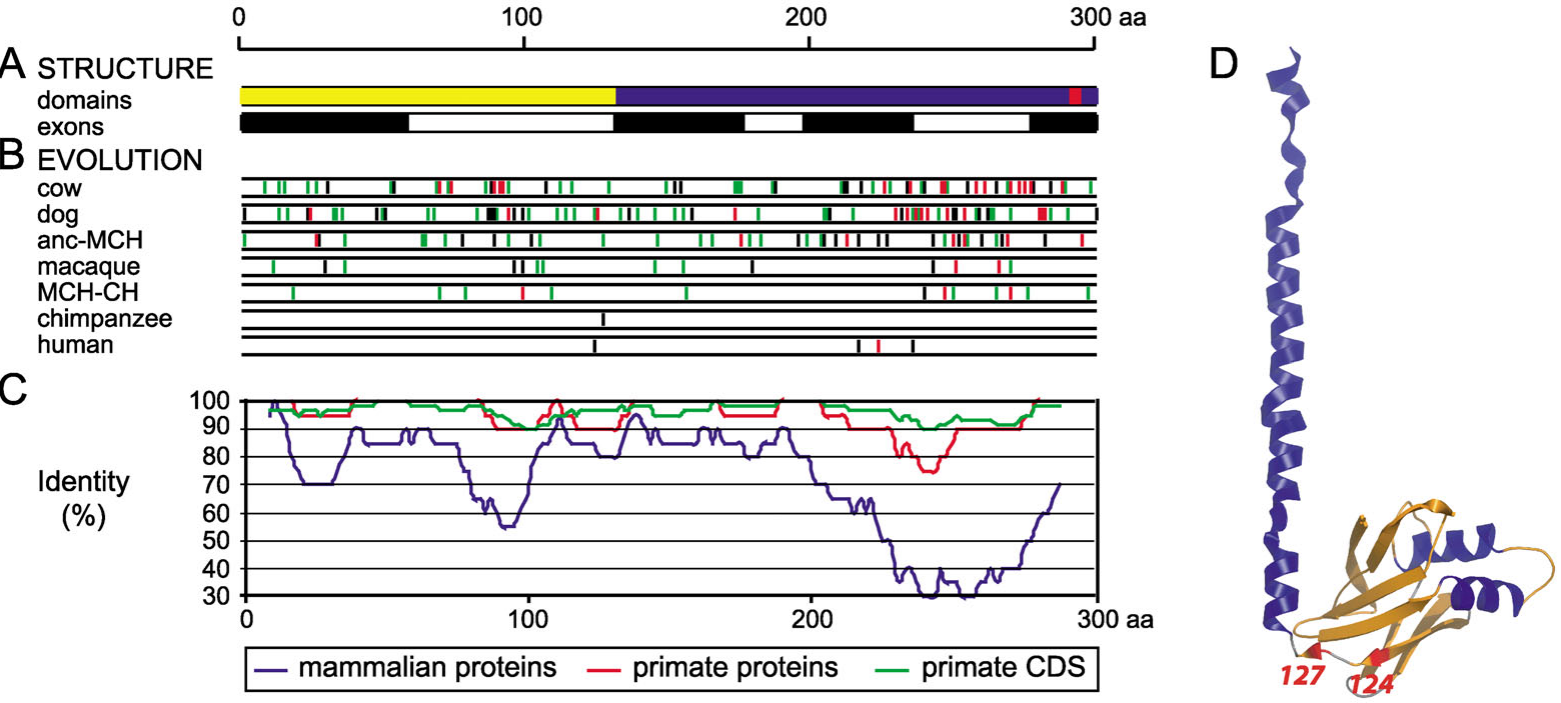
**Structure and evolution of Cernunnos-XLF proteins inmammals**. The scale of all plots corresponds to the protein alignment of 299 amino acids, and positions in the coding sequence (CDS) were scaled accordingly. **A**. Structure of human Cernunnos-XLF CDS and protein. The first scheme shows the positions of the major predicted domains in the Cernunnos-XLF protein [after ref. 5]. The N-terminal globular head domain is marked in yellow, the remaining coiled-coil structure in blue, the putative nuclear localization signal is in red. The second scheme shows the positions of the coding exons (2–8) in the CDS (the odd exons are black and the even ones are white). **B**. CDS substitutions during evolution. The expected ancestral coding sequence was estimated using maximum likelihood codon reconstruction implemented in PAML. Nonsynonymous/synonymous (ω = Ka/Ks) ratios were free to vary in all branches. Positions marked in green correspond to synonymous changes in a given lineage. Bars representing nonsynonymous changes are black if conservative, red if nonconservative (see methods). "MCH-CH" corresponds to the ancestral lineage between the common ancestor of macaque, chimpanzee, and human (MCH) to the common ancestor of human and chimpanzee (CH), "anc-MCH" represents the lineage from the common ancestor of all taxons to MCH (see Fig. 2). **C**. Conservation at the nucleotide level in primates, and protein level in primates and mammals. The Y axis corresponds to the proportion of conserved (identical) positions in the CDS (a 60-bp overlapping window and 6-bp steps) and the protein alignment (window 20-aa, step 2-aa). **D**. Predicted structure of the Cernunnos-XLF protein. The structure for the first 185 aa was predicted by structural alignment to XRCC4 (see methods). The red parts highlight positions that are different between human and chimpanzee, aa 124 changed in the human lineage, aa 127 in the chimpanzee branch. Three other positions were changed in humans – aa 216, 223, 235 (see Fig. 2B).

Comparison of individual mammalian copies revealed a variable rate of amino acid replacements along *Cernunnos-XLF *(Fig. 1). While synonymous changes are dispersed relatively uniformly, nonsynonymous changes are clustered in several domains (Fig. 1B,C). Most variable is the C-terminal part between aa 212–281. Another less pronounced variable region is at aa positions 87–99. This profile is similar to the protein conservation in vertebrates [5]. There are five nonsynonymous substitutions between the human and chimpanzee genes (four of them seem to be human-specific) and no synonymous changes. Interestingly, these five changes are unevenly distributed along the protein. Position 124, which changed in the human lineage, and the chimpanzee substitution at aa 127 are located within a conserved linker between the N-terminal globular head domain and the remaining coiled-coil part (Fig. 1D). Three other positions 216, 223, and 235 changed in humans, and cluster within the predicted end of a coiled-coil C-terminal domain [Figure 1SA in ref 5].

### Adaptive evolution of *Cernunnos-XLF *genes in the human lineage

**Figure 2:**
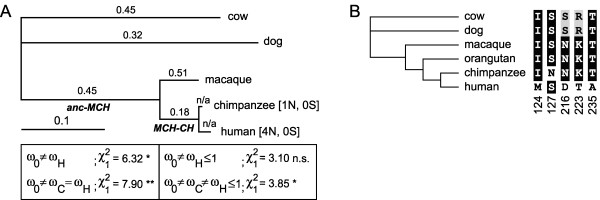
**Phylogenetic tree and Ka/Ka ratio forCernunnos-XLF coding sequences**. **A**. Phylogenetic tree and Ka/Ka ratio for Cernunnos-XLF mammalian coding sequences. Phylogenetic tree obtained by the free ratio codon model in PAML. The ancestral branches from Fig. 1A are indicated by italics. Branch labels mark the ω = Ka/Ks ratios for corresponding branches. For the human and chimpanzee branches we could not calculate the Ka/Ks ratio and instead we list the number of synonymous (S) and nonsynonymous (N) changes in square brackets. The boxes list selected tested hypotheses. The Ka/Ks rate is designated as ω_H _for the in the human lineage, ω_C _for the chimpanzee lineage, and ω_0 _for all other lineages. A single asterisk indicates *P *< 5%, χ^2^_1 _= 3.84, double asterisk indicates *P *< 1%, χ^2^_1 _= 6.63. The left box tests the hypotheses that the Ka/Ks ratio for the human lineage is the same as for the rest of the tree (rejected at *P *< 5%), and that both human and chimpanzee lineages have the same Ka/Ks ratio shared with other branches (rejected at *P *< 1%). The right box shows tests Ka/Ks ≤ 1 for the human lineage (not significant) and for both human and chimpanzee lineages (rejected at *P *< 5%). **B**. Amino acid residues for five critical positions changed between human and chimpanzee. The tree also includes orthologous positions from the orangutan Cernunnos-XLF protein. The figure shows conservation of the critical positions in macaque and orangutan, which represents the most likely ancestral state. Four human and one chimpanzee changes indicated in the figure represent the most parsimonious scenario of *Cernunnos-XLF *evolution.

The analysis of individual branches in the phylogenetic tree (Fig. 2) revealed signs of negative selection (Ka/Ks < 1) on most branches, but the presence of five nonsynonymous and the lack of synonymous substitutions indicate possible positive Darwinian selection in humans and chimpanzees. Indeed, likelihood ratio tests confirm that the human and possibly also chimpanzee lineages evolved under different Ka/Ks rates compared to the rest of the tree (significant; Fig. 2). These results are robust even when one by one we discarded all individual changes (not shown). When both human and chimpanzee lineages were combined into one group, the resulting joined Ka/Ks ratio is above 1 (borderline significant) suggesting positive selection. Therefore, we can conclude that the *Cernunnos-XLF *locus evolved adaptively under positive selection in humans. Whether chimpanzees also experienced positive selection is unclear, but the rate of protein evolution seems to be lower compared to humans. Finally, we were also interested in how *Cernunnos-XLF *evolves in the recent human population. HapMap data indicates the lack of recent positive selection on *Cernunnos-XLF *[16]. However, given the presence of two nonsynomous and no synonymous polymorphic positions in the human population [17] we cannot rule out that some positive selection still operates on this locus.

As mentioned above, the amino acid replacements in the human and chimpanzee lineages are clustered and, as a consequence, adaptive evolution in *Cernunnos-XLF *appears to be concentrated in very specific regions. One hotspot is located in the region between the predicted N-terminal globular head domain and the long coiled-coil part (Fig. 1D). The second rapidly evolving region is located at the putative C-terminal end of the coiled-coil domain (not shown). The exact structure and function of these regions in Cernunnos-XLF is unknown, but in the case of the structurally similar XRCC4 protein, the head domain seems to interact with DNA and/or proteins while the coiled-coil region binds the linker connecting two BRCT repeats of ligase IV [18-20]. It is tempting to speculate that the adaptive evolution around the coiled-coil region is related to a putative interaction of this region with ligase IV and by extension to the proposed Cernunnos-XLF function: promoting the DNA ligation function of the XRCC4-ligase IV complex [4,5].

### *Cernunnos-XLF *– another factor in human brain expansion?

Genome-wide comparisons have revealed that a significant number of protein-coding genes undergo adaptive evolution in humans [17,21,22]. Notably, the dramatic increase in brain size and complexity during human evolution was accompanied by accelerated, often positive, selection on several genes involved in regulation of brain size and the nervous system in general [7-15]. These genes include two primary microcephaly loci under strong positive selection in humans *ASPM *(abnormal spindle-like), and *microcephalin/MCPH1*; and possibly also other microcephaly-associated loci with an increased Ka/Ks rate in primates *PAFAH1B1 *(alpha subunit of platelet-activating factor acetylhydrolase 1B) and *SHH *(sonic hedgehog), although the latter two may be merely under relaxed constraints [12]. Adaptive evolution of *Cernunnos-XLF *thus fits the general pattern of simultaneous selection acting upon several microcephaly-associated genes in humans.

How can the Cernunnos-XLF function in nonhomologous end-joining contribute to our brain size? It seems natural to assume that brain expansion should reflect an increased number of cells, and thus cell divisions during brain neurogenesis [15,23]. A direct extrapolation of this assumption is that the increased brain size could be achieved by an increased efficiency of factors involved in cell cycle progression, mitosis, or by preventing apoptosis. Consistent with this hypothesis are cellular functions of two strongly selected primary microcephaly genes *ASPM *and *microcephalin*. ASPM is a mitotic spindle protein that may participate in regulation of cell division during neurogenesis [24]. *Microcephalin *encodes a DNA damage response protein regulating the BRCA1-CHK1 DNA damage response pathway [25,26]. This suggests that *microcephalin*-linked primary microcephaly is related to cellular checkpoint defects causing increased cellular apoptosis in neural lineages [26]. Therefore, effective repair of DNA damage at cellular checkpoints is a prerequisite for efficient cell proliferation during neurogenesis, and adaptive evolution of *microcephalin *may reflect this requirement.

It appears that both functional homologous recombination and nonhomologous end-joining (NHEJ) are essential during nervous system development. Inactivation of some NHEJ components, including ligase IV and XRCC4, in mouse causes apoptosis of post-mitotic neurons [27]. As a consequence, positive selection on Cernunnos-XLF may be related to the essential role of this factor in efficient DNA damage repair by NHEJ and, in turn, in preventing apoptosis in neuronal progenitors.

In summary, adaptive evolution of *Cernunnos-XLF *in humans fits into the broader scheme of microcephaly gene evolution in primates. On one hand, each positively selected gene operates at a different level: the spindle protein ASPM on the level of cell division, microcephalin by participating in DNA damage response during cellular checkpoints, and Cernunnos-XLF by direct involvement in NHEJ repair of damaged DNA. On the other hand, the phenotypic effect is similar – an increased number of neurons in the developing brain by either efficient cell proliferation (presumably in the case of ASPM) or prevention of apoptosis (microcephalin, Cernunnos-XLF).

While association of *Cernunnos-XLF *selection with increased brain size is attractive in the context of simultaneous adaptation of several brain size determinants, there are also other possible explanations. Cernunnos-XLF deficiency is manifested by an increased susceptibility to infections due to immunodeficiency caused by impaired renewal of T and B cells [4]. Delayed reproduction in humans may require a highly efficient immune system that is able to fight infections during the prolonged pre-reproductive period of life. Another possibility is increased pressure on the general tumor suppression function of DSB repair in humans due to differences in reproductive cycle, changes in diet, lifestyle and/or exposure to mutagenic agents. Given its essential role in NHEJ, Cernunnos-XLF deficiencies may be associated with an increased cancer risk [4,5]. Indeed, the tumor suppressor BRCA1 is another, well studied DNA repair factor under positive selection in humans [28,29]. Moreover, tumor suppressor genes in general seem to evolve under higher Ka/Ks rate in humans [22]. While several possible explanations are possible, it is clear that the complete elucidation of *Cernunnos-XLF *evolution in humans will require better understanding of Cernunnos-XLF function and its impact on various cellular processes.

## Conclusion

Cernunnos-XLF is a new component of the nonhomologous end-joining machinery mutated in human immunodeficiency with microcephaly [4,5]. Using newly obtained coding sequences in chimpanzee and rhesus macaque as well as dog and cow orthologs, we reconstructed the evolutionary history of *Cernunnos-XLF *in mammals. We found that *Cernunnos-XLF *is under positive selection in the human lineage. Hotspots of adaptive evolution are concentrated around the putative DNA ligase IV binding domain. After *ASPM *and *microcephalin*, *Cernunnos-XLF *is the third identified microcephaly-associated locus under strong adaptive evolution in humans and possibly played a role in the expansion of brain size in humans. We speculate that Cernunnos-XLF may contribute to the increased number of brain cell in humans by efficient double strand break repair, which helps to prevent frequent apoptosis of neuronal progenitors and aids mitotic cell cycle progression.

## Methods

### Reconstruction of the macaque and chimpanzee Cernunnos-XLF coding sequence

**Figure 3:**
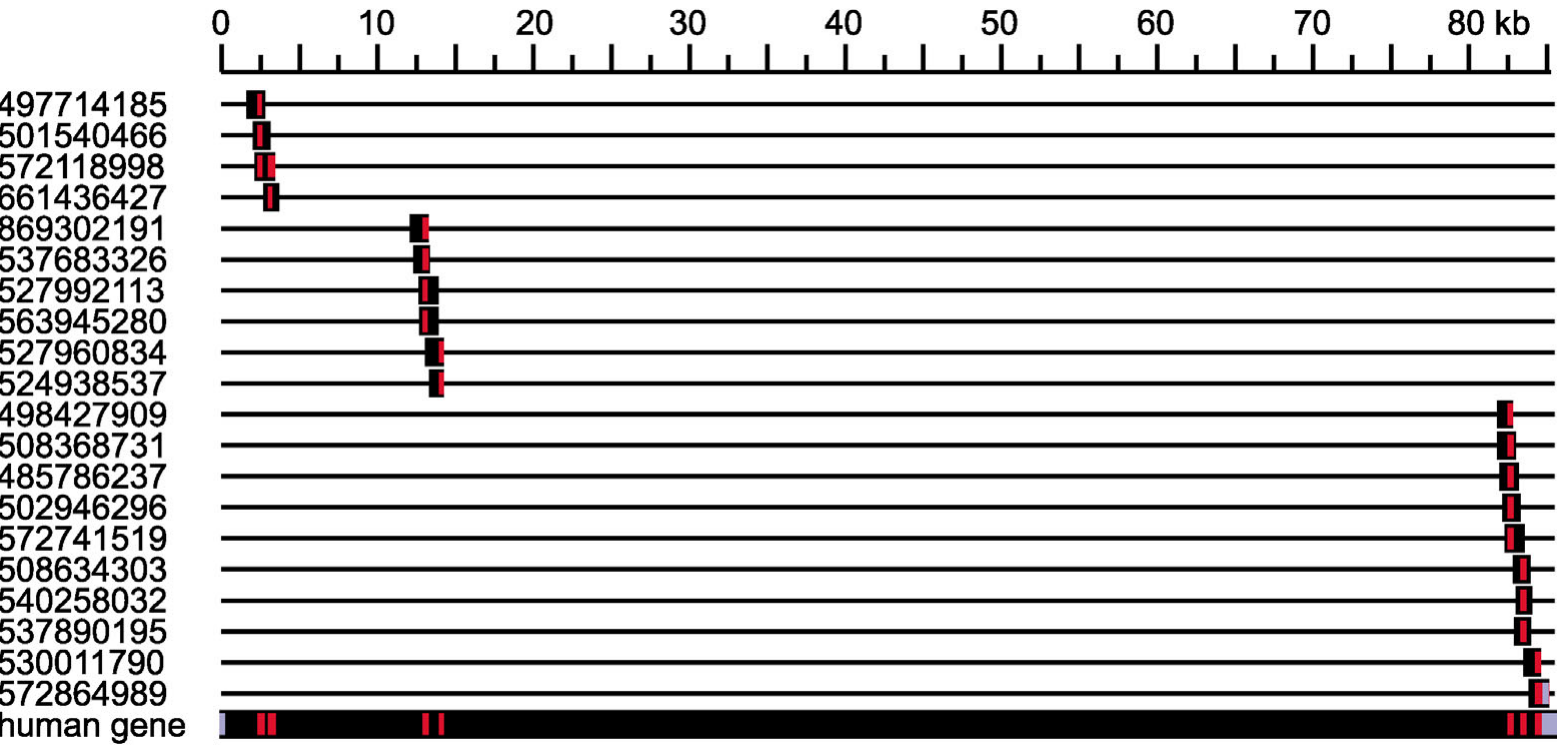
**Reconstruction of rhesus macaque coding sequences**. Figure shows positions of sequences from the macaque whole genome shotgun trace archive (Macaca mulata WGS) aligned on the human *Cernunnos-XLF *gene. Coding exons are in red, noncoding parts of exons in blue, introns in black. The numbers on the left correspond to sequence identifiers from the trace archive.

We used human coding sequence (CDS) as a probe for discontiguous Mega BLAST [30] searches against the macaque whole genome shotgun trace archive (Macaca mulata WGS). For all highly similar hits in the trace archive, the full-length trace sequences were aligned using BLAT [31] to the human *Cernunnos-XLF *gene, including introns, to ensure proper localization. The consensus sequence obtained from the alignment of individual trace sequences represents the expected macaque Cernunnos-XLF coding sequence. The predicted macaque CDS was covered by two or more sequences from the trace archive along its complete length (Fig. 3). The chimpanzee *Cernunnos-XLF *gene was obtained from BLAT [31] alignment of the human copy with the chimpanzee genome assembly, and the coding sequence homologous to human CDS was extracted.

### Sequence analysis

Mammalian Cernunnos-XLF protein sequences were aligned using Dialign2 [32] and the alignment was visualized in GeneDoc [33]. Synonymous and nonsynonymous substitutions were obtained using SNAP [34]. Gonnet PAM250 matrix [35] was applied to classify substitutions as conservative or non-conservative. We considered changes to be conservative if the score was > 0.5. We used ancestral sequence reconstruction and the free ratio codon model in PAML v. 3.13 [36] to reconstruct phylogeny and estimate placement of substitutions along individual branches of the phylogenetic tree. The phylogenetic tree was drawn in TREEVIEW [37].

### Detection of positive selection

Positive selection along individual branches was detected by likelihood ratio tests as described previously [38]. First, we compared the log-likelihood value for one-ratio and two-ratio models to detect possible different Ka/Ks ratios in individual lineages. To test whether these lineages evolve with Ka/Ks significantly >1, we compared the two ratio models with the Ka/Ks ratio set to 1 and with free (estimated) Ka/Ks for the lineages under consideration.

### Prediction of the protein structure

Structural alignment of human Cernunnos-XLF protein to the DNA repair protein XRCC4 (1fu1) was performed using 3D-PSSM [39] and SWISS MODEL [40] servers, analogously to ref [5]. The predicted structure of the human Cernunnos-XLF protein was visualized in PyMOL [41].

## Abbreviations

DSB – double strand break

HR – homologous recombination

NHEJ – nonhomologous end-joining

CDS – coding sequence

## Authors' contributions

AP conceived the study and performed the data analysis. AP and JJ wrote the paper. Both authors read and approved the final manuscript.

## Reviewers' comments

### Reviewer's report 1

*Chris Ponting, Department of Human Anatomy and Genetics, South Parks Road, Oxford OX1 3QX, UK, with additional advice from Richard Emes, Department of Biology, Darwin Building, University College London, Gower Street, London, WC1E 6BT, UK*.

There is much interest in identifying nucleotide substitutions that might underlie human-specific biology. Pavlicek & Jurka have undertaken an evolutionary analysis of Cernunnos-XLF and propose that this gene has experienced positive selection of one or more nonsynonymous nucleotide substitutions. As this gene is mutated in individuals with microcephaly, the authors propose a causative link between brain enlargement and Cernunnos-XLF adaptive evolution.

Pavlicek & Jurka base their proposal of adaptive evolution in chimpanzee and human Cernunnos-XLF upon 5 inferred nucleotide substitutions, all of which are proposed to have been nonsynonymous. The major issue in the authors' conclusion of positive selection is whether the extremely short branch lengths of chimpanzee and human sequences affect predictions. For example, approximately 20% of chimpanzee/human divergence is due to substitutions that are not fixed. If any one of the 5 substitutions were discarded, would the significance of these findings remain? Similarly, if the chimpanzee sequence were to be discarded would the predictions still hold?

**Author response**: *We agree with the reviewers that some substitution(s) in the human/chimpanzee lineages may in fact be polymorphic mutations. Therefore, following the reviewers' advice, for each of the five nonsynonymous substitutions, we replaced the particular (changed) position by its ancestral (unchanged) codon. We concentrate initially on the increased Ka/Ks ratio in the human lineage, which seems to be under the strongest positive selection:*

**Table 1:** 

**Change tested**	***Single rate *ω_*0 *_= ω_*H *_*(*ℓ_*0*_*)***	***Two rates *ω_*0 *_≠ ω_*H *_*(*ℓ_*1*_*)***	***2(*ℓ_*1*_-ℓ_*0*_*)***
*hum124*	*-2167.41*	*-2165.02*	4.78*
*chimp127*	*-2166.41*	*-2163.24*	6.34*
*hum216*	*-2168.82*	*-2166.44*	4.76*
*hum223*	*-2166.84*	*-2164.46*	4.76*
*hum235*	*-2168.50*	*-2166.12*	4.76*
*no change*	*-2174.88*	*-2171.72*	6.32*

* *Significant at P < 5%*, χ^2^_1 _= *3.84*

As expected, when we discarded individual human-specific changes, the likelihood ratio difference decreased, from 6.32 to 4.76–4.78, but remained significant. The removal of the chimpanzee-specific substitutions at position 127 had negligible effect on the significance of the human Ka/Ks ratio. The same applies for the test of difference between human and chimpanzee lineages versus the rest of the tree:

**Table 2:** 

**Change tested**	***Single rate *ω_*0 *_= ω_*C *_= ω_*H*_*(*ℓ_*0*_*)***	***Two rates *ω_*0 *_≠ ω_*C *_= ω_*H*_*(*ℓ_*1*_*)***	***2(*ℓ_*1*_-ℓ_*0*_*)***
*hum124*	*-2167.41*	*-2164.23*	6.36*
*chimp127*	*-2166.41*	*-2163.24*	6.34*
*hum216*	*-2168.82*	*-2165.65*	6.34*
*hum223*	*-2166.84*	*-2163.67*	6.34*
*hum235*	*-2168.50*	*-2165.33*	6.34*
*no change*	*-2174.88*	*-2170.93*	7.9**

*We can see that the results on unequal ω = Ka/Ks ratios are robust for removal of all individual positions*.

*We cannot discard the chimpanzee sequence, because this sequence is crucial in defining human-specific changes. When we discarded the chimpanzee copy, the Ka/Ks ratio was not significantly different from the rest of tree. However, in this test we did not analyze Ka/Ks in the human lineage, but the long lineage from the common ancestor of human and macaque to modern humans. Figure *2*(now 2A) shows that most of the time this lineage was under negative selection*.

It is probable that the issue of resolving power at the branch tips could be resolved by additional information, particularly of additional ape orthologous sequences.

**Author response**: *We agree, but we could not reconstruct other full-length primate orthologs, as sequence traces are incomplete. The most complete is the orangutan Cernunnos-XLF copy, which lacks only one exon. However, since the only difference between human and chimpanzee are five nonsynonymous changes, we decided to concentrate on these five positions (newly added Fig *2B). *The figure shows conservation of the critical positions in macaque and orangutan and represents the most likely ancestral state. Four human and one chimpanzee changes indicated in the figure represent the most parsimonious scenario of Cernunnos-XLF evolution. Positions 124, 127 are covered by four trace sequences (764029532, 871856388, 853589440, and 799919414) and positions 216, 223, and 235 by one sequence (850346752)*.

*We would like to stress that all five changes between human and chimpanzee would be nonsynonymous no matter what method we use (supposing that the genomic sequences are correct). The only question is in which lineage they occurred. This additional orangutan data gave us confidence that reconstruction of the human-chimpanzee ancestral sequence was correct and that there is a high probability that four changes occurred in the human lineage and only one in chimpanzees*.

Additionally, haplotype analysis of Cernunnos-XLF would be required to investigate whether positive selection has been ongoing in more recent times. Results from these approaches would have been appropriate to bolster the authors' proposal.

**Author response**: *The reviewers (as well as one other reviewer, see below) raised an excellent point. Although we were primarily interested in the signs of positive selection in the last 5–6 million years of human evolution, it is interesting to check for positive selection on Cernunnos-XLF in more recent times. As suggested by another reviewer, we used the recent study by Voight et al. (PLoS Biology 2006 4:e72) to evaluate positive selection in the recent human population. Voight et al. used the HapMap data to detect signatures of positive selection in three different populations: east Asians (ASN), western Europeans (CEU), and sub-Saharan Africans (Yoruba – YRI). The authors set up a public web server Haplotter **that analyzes HapMap data for all human chromosomes and also for individual genes. Based on this dataset, it seems that Cernunnos-XLF is not under positive selection in recent human populations (the empirical p-values are 0.25 for CEU, 0.41 for YRI, and 0.999 ASN)*.

*However, we would like to point out some limitations of the HapMap data. First of all is the time limitation: it seems that favored haplotypes are roughly just 6,600 and 10,800 years old for African and non-African populations, respectively (Voight et al. 2006). The application of haplotype analysis for detection of selection in longer periods is limited. Indeed, in many cases there is a low correspondence between positive selection detected from interspecies analysis and analysis of human polymorphism. For example, a well documented example of a strongly selected gene in the human lineage BRCA1 (Ka/Ks > 2.5, Pavlicek et al. 2004 HMG 13:2737-51.) does not show any sign of positive selection in the recent population (Haplotter, the empirical p-values are 0.15, 0.61, and 0.999). More surprisingly, even ASPM and microcephalin, which are both known to be under selection in the recent human population (Mekel-Bobrov et al. 2005 Science 309:1720-2; Evans et al. 2005 Science 309:1717-20), do not show any significant selection from HapMap data either (ASPM: the p-values are 0.999 for CEU, 0.23 for YRI, and 0.54 for ASN; microcephalin: the p-values are 0.55, 0.17, 0.54). This discrepancy is probably related to data selection (resequencing of 89 individuals versus public HapMap data) and also high variability in the strength of selection between different human populations*.

*Interestingly, Bustamante et al. 2005 (Nature 437:1153-7.) found two nonsynonymous and no synonymous polymorphic positions within the human population. Thus, it is possible that Cernunnos-XLF is still under selection in the human population (despite the lack of support from the haplotype data). Therefore we added two sentences: "HapMap data indicates the lack of recent positive selection on Cernunnos-XLF *[16]. *However, given the presence of two nonsynomous and no synonymous polymorphic positions in the human population *[17]*we cannot rule out that some positive selection still operates on this locus."*

Also, it is unclear why the authors have not taken advantage of the mouse and rat genome sequences, or pig and opossum ESTs, or even the unassembled sequences from rabbit, armadillo and elephant, which are provided from the UCSC's genome browser site. Would consideration of these sequences provide evidence to support, or otherwise, the authors' prediction?

**Author response**: *When we added mouse, rat, pig, and possum Cernunnos copies, the statistical significance of the likelihood tests increased, not decreased. For instance, the test for an increased Ka/Ks rate in the human lineage yielded chi-square 6.78 (1 d.f.; p < 0.05) up from 6.32 (p < 0.05). The chi-square test for different Ka/Ks in the human and chimpanzee lineage increased to 8.38 (p < 0.01) from 7.90 (p < 0.05). Apart from human and chimpanzee, all the Ka/Ks for all other lineages is < 1; 0.52 for mouse, 0.54 (rat), 0.27 (pig), and 0.25 (possum). Therefore, our results are more robust after addition of more mammalian sequences. Since the crucial part for our analysis is the primate part of tree, we decided not to include these new sequences in the analysis. Non-primate sequences merely serve as outgroups, and for this purpose cow and dog sequences are sufficient*.

I would also advise a greater degree of scepticism in the manuscript. Causal relationships between microcephaly genes, their proposed adaptive evolution and brain enlargement cannot yet be accepted without the consideration of other possible explanations. For example, as with other microcephaly genes, Cernunnos-XLF is expressed widely and is not brain-specific (indeed its expression in the brain is not obviously elevated relative to other tissues). The chimpanzee brain appears not to have enlarged greatly since the last common ancestor with humans, and the three-fold enlargement of our brains only occurred in the last 3 million years. Timing initiation of Cernunnos-XLF adaptive evolution relative to physiological innovations would provide greater insights into these causal relationships than this manuscript can yet provide.

**Author response**: *We agree with reviewers that the role of Cernunnos-XLF in human brain expansion is speculative. In the last paragraph of the Discussion we wrote that: "While association of Cernunnos-XLF selection with increased brain size is attractive in the context of simultaneous adaptation of several brain size determinants, there are also other possible explanations." Two such explanations are mentioned in the same paragraph. In the Abstract, we clearly stated that Cernunnos-XLF "possibly played a role" in human brain expansion. So far, little is known about its function. However, the involvement of another positively selected candidate gene microcephalin/MCPH1/BRIT1 in DNA damage response (Lin et al, 2005) indicates that efficient DNA repair can be crucial in early brain development. In this context, the proposed selection on the repair factor Cernunnos-XLF is speculative, but in line with some current proposals on the mechanisms of human brain expansion*.

*Concerning the selection on chimpanzees, the mode of chimpanzee evolution is unclear. Given the single change encountered after the split with humans we cannot conclude if the chimpanzee locus is under selection or not. For this reason we used two tests that include and exclude the chimpanzee branch from positively selected lineages. Clearly the human lineage was under positive selection, and this result is robust even when we considered the human lineage alone from the rest of the tree including the chimpanzee branch (Figure *2*). The fact that the human lineage, not chimpanzee, exhibits a higher nonsynonymous rate is in fact in agreement with human, not chimpanzee, brain enlargement. It seems that our description was confusing; we changed the corresponding paragraph of Results to clearly state that the major part of positive selection happened in humans and is significant by itself. In the Abstract we speak only about adaptive evolution in humans*.

#### Other comments

p3 "Interestingly, these five changes are nonrandomly distributed along the protein." Either apply a statistical test or delete.

**Author response**: *"Nonrandomly" was replaced by more accurate "unevenly", but we prefer to keep that sentence in the text, because it points out potential hotspots of adaptive evolution and interesting regions for functional studies*.

p4 The Dorus et al. findings (11) relevant to SHH and PAFAH1B1 do not conclusively show that positive selection, as opposed to relaxed constraints, for example, has occurred. This should be made clear.

**Author response**: *We agree and the sentence was changed*.

p4–5. In the summary, there is no caveat that these genes might not, after all, have evolved adaptively due to brain enlargement.

**Author response**: *We agree. The last paragraph before Conclusions clearly indicates that other explanations are possible (e.g. "While several possible explanations are possible, it is clear that the complete elucidation of Cernunnos-XLF evolution in humans will require better understanding of Cernunnos-XLF function and its impact on various cellular processes"). Also in the Conclusions we clearly state "Cernunnos-XLF ... possibly played a role in the expansion of brain size". A similar sentence is used in the Abstract*.

(5) It is unclear whether Figure 3 is required.

**Author response**: *The macaque coding sequence is crucial for estimating the ancestral state before the split of humans and chimpanzees and therefore we prefer to include it in some form in the manuscript. The figure can be moved to a supplement, but since the manuscript is very short (and the journal is electronic), we decided to keep Figure *3* in the main text*.

### Reviewer's report 2

Kateryna Makova (assisted by Erika Kvikstad), Department of Biology, 518 Mueller Lab, Penn State University, University Park, PA 16802

The manuscript by Adam Pavlicek and Jerzy Jurka contributes new information to the list of human genes evolving under positive selection. The authors examined a locus (Cernunnos-XLF) associated with microcephaly because of the evidence that other genes linked to this disorder have evolved under adaptive evolution in humans (ASPM and microcephalin). They used comparative sequence information for this locus from several mammalian species and identified an excess of nonsynonymous substitutions occurring in the human lineage (after divergence from the common ancestor with chimpanzee), which is suggestive of positive selection. Four nonsynonymous substitutions occurred in the human lineage, as compared with only one such substitution in the chimpanzee lineage.

This result is of great interest particularly in the light of the ongoing (and difficult!) quest for genes that make us humans, so publication of this manuscript in *Biology Direct *is recommended. However, an additional analysis would strengthen the conclusions made by the authors.

1. It would be informative to see the results of other testing of the hypothesis of selection acting on this locus. For instance, one could incorporate human polymorphism data available from the HapMap project and search for potentially reduced levels of heterozygosity in the region. Such data are freely available and could also provide information on the timing of selective constraints in the region (is it restricted to modern humans?). Is Cernunnos-XLF among the loci showing a signature of strong recent positive selection in the study by Voight et al. (PLOS Biology, 2006), which utilized HapMap data? (We realize that the study by Voight et al. just came out and thus could not have been included by the authors in the original draft.)

**Author response**: *The reviewer (as well as other reviewers, see above) raised an excellent point. Although we were primarily interested in the signs of positive selection in the last 5–6 million years of human evolution, it is interesting to check for positive selection on Cernunnos-XLF in more recent times. As suggested by the reviewer, we used the recent study by Voight et al. (PLoS Biology 2006 4:e72) to evaluate positive selection in the recent human population. Voight et al. used the HapMap data to detect signatures of positive selection in three different populations: east Asians (ASN), western Europeans (CEU), and sub-Sahara Africans (Yoruba – YRI). The authors set a public web server Haplotter ** that analyzes HapMap data for all human chromosomes and also for individual genes. Based on this dataset, it seems that Cernunnos-XLF is not under positive selection in recent human populations (the empirical p-values are 0.252749 for CEU, 0.408690 for YRI, and 0.999954 ASN)*.

*However, we would like to point out some limitations of the HapMap data. First of all is the time limitation, it seems that favored haplotypes are roughly just 6,600 and 10,800 years old for African and non-African populations, respectively (Voight et al. 2006). The application of haplotype analysis for detection of selection in longer periods is limited. Indeed, in many cases there is a low correspondence between positive selection detected from interspecies analysis and analysis of human polymorphism. For example, a well documented example of a strongly selected gene in the human lineage BRCA1 (Ka/Ks > 2.5, Pavlicek et al. 2004 HMG 13:2737-51.) does not show any sign of positive selection in the recent population (Haplotter, the empirical p-values are 0.148074, 0.607928, and 0.999954). More surprisingly, even ASPM and microcephalin, which are both known to be under selection in the recent human population (Mekel-Bobrov et al. 2005 Science 309:1720-2; Evans et al. 2005 Science 309:1717-20), do not show any significant selection from HapMap data either (ASPM: the p-values are 0.999955 for CEU, 0.225666 for YRI, and 0.539616 for ASN; microcephalin: the p-values are 0.547812, 0.171512, 0.544621). This discrepancy is probably related to data selection (resequencing of 89 individuals versus public HapMap data) and also a high variability in the strength of selection between different human populations*.

*Interestingly, Bustamante et al. 2005 (Nature 437:1153-7.) found two nonsynonymous and no synonymous polymorphic positions within the human population. Thus it is possible that Cernunnos-XLF is still under selection in the human population (despite the lack of support from the haplotype data). Therefore we added a sentence "HapMap data indicates the lack of recent positive selection on Cernunnos-XLF *[16]. *However, given the presence of two nonsynomous and no synonymous polymorphic positions in the human population *[17]*we cannot rule out that some positive selection still operates on this locus."*

2. The authors cite a publication by Bustamante, CD et al (Science, 2005) which investigates >11,000 human genes for signatures of selection and includes both the comparative analysis between human and chimpanzee and analysis of polymorphisms in humans. It would be interesting to see whether Cernunnos-XLF was included in Bustamante et al.'s analysis and, if it was, where it ranks in comparison with other genes.

**Author response**: *Bustamante et al. 2005 used the McDonald-Kreitman test to evaluate natural selection on human genes. This test (in Bustamante et al. 2005) did not yield any significant support for positive selection on Cernunnos-XLF. However, we should note that the McDonald-Kreitman test has some limitations. Positive selection is detected as an excess of nonsynonymous/synonymous divergence (fixed changes between species) compared to nonsynonymous/synonymous polymorphism (in our case human Cernunnos-XLF polymorphism). However, if the positive selection still operates on the population level, the difference between different species vs. within population may not be significant, yet the locus is under positive selection. For instance, microcephalin/MCPH1 in the Bustamante et al. dataset is detected to be under negative selection "at 95% credibility level", although there is solid evidence that this gene was under positive, not negative, selection in the recent population (Evans et al. 2005 Science 309:1717-20). We could not compare ASPM data, as it is present twice (under different Refseq names) in the dataset and both copies analyzed are severely truncated*.

*Bustamante et al. found two nonsynonymous and no synonymous polymorphic Cernunnos-XLF positions within the human population, so it is possible that the gene is still under selection in the human population (despite the lack of support from haplotype data) and thus the McDonald-Kreitman is unable to detect any difference. In conclusion, due to the incompatible methods used, we cannot directly compare our results with the study of Bustamante et al. 2005; as the two studies asked different questions*.

### Reviewer's report 3

Gáspár Jékely, European Molecular Biology Laboratory, Developmental Biology Unit, Meyerhofstrasse 1, 69117 Heidelberg, Germany

The authors identified signs of positive selection during human evolution in the Cernunnos protein. It is interesting given that certain mutations in the human gene lead to microcephaly. The authors speculate that positive selection in the gene may have contributed to increased brain size evolution in humans.

However, as discussed in the paper, loss of Cernunnos activity also leads to immunodeficiency in humans and it is equally possible that positive selection acted to modify immune functions during primate evolution. To decide between these two possibilities it may help to check in large-scale comparative expression datasets (e.g. Science 5566:340-3) whether expression levels of components of the XRCC4-Ligase IV complex changed in humans in the brain.

**Author response**: *XRCC4 and DNA ligase IV were not included in the study of Enard et al. 2002 (Science 296(5566):340-3). We therefore decided to look at similar studies. Caceres et al. 2003 (PNAS 100:13030-5) used Affymetrix HG-U95Av2 array to detect differential expression in human, chimpanzee, and rhesus macaque brains. Although the full dataset from that study is not available, a supplementary table listing mRNA with significantly different levels between species does not contain transcripts encoding ligase IV or XRCC4. Since the HG-U95Av2 array contains ligase IV (probe-set id 963_at) and XRCC4 (1360_at) transcripts, we conclude that these two genes are not differentially expressed in human and chimpanzee brains. We also analyzed data from a more recent study by Khaitovich et al. 2004 (GR 14:1462-73). The supplementary set contains expression data for ligase IV. While this gene seems to be more highly expressed in the human brain, especially in the primary visual cortex, anterior cingulate cortex, cerebellum, and Broca's area, none of these results is significant when using the author's criteria*.

*Since the available data do not provide conclusive data about ligase IV overexpression in humans, we did not include any comment in the manuscript. However, we would like to point out that both increased expression or higher efficiency/fidelity of Cernunnos-XLF proteins in DSB repair may have a similar effect on the cellular level and contribute to brain expansion. If the former were true as the reviewer suggested, we would expect more changes in regulatory regions (promoter, enhances, not detected), for the latter we would expect protein changes, such as those we have described in our manuscript*.

The proposed scenario for brain size increase only works if double stranded break repair is limiting during brain development. Is actually apoptosis regulated at the level of double-strand break repair? In other words even if impaired double stranded break repair can lead to apoptosis it may not mean that its increased activity can prevent it.

**Author response**: *We believe that there is very strong evidence supporting the fact that double-strand break (DSB) repair is one of the most important anti-apoptotic factors during brain development. It is known that neuronal cells are one of the most sensitive cells to deficient double-strand break (DSBs) repair. For example, ligase IV and XRCC4 knockouts are lethal due to massive neuronal apoptosis and it seems that "neurons strictly require the XRCC4 and DNA ligase IV end-joining proteins" (Gao et al. 1998 Cell 95:891-902; see also Barnes et al. 1998 Curr Biol. 8:1395-8; Lee et al. 2000 Genes Dev. 14:2576-80). Moreover, it has been demonstrated that neuronal apoptosis in ligase IV- (and thus NHEJ-) deficient cells requires the general DNA damage-signaling factor ATM (Lee et al. 2000 Genes Dev. 14:2576-80). ATM is the key component in the DSB signaling pathway that triggers cell cycle arrest and possibly also apoptosis (via p53 phosporylation) at all stages of cell cycle checkpoint (Rich et al. 2000 Nature 407:777-83; Kastan & Bartek 2004 Nature 432:316-23). During the G1/early S phase homologous recombination is suppressed and DSB repair mostly relies on error-prone NHEJ (e.g. Rothkamm et al. 2003 Mol Cell Biol. 23:5706-15). Therefore, more efficient (or better regulated) NHEJ can limit DNA damage by DSBs and in turn suppress the ATM signaling pathway, especially during the G1 and G1/S cell-cycle checkpoints, which potentially leads to cell death*.

### Reviewer's report 4

Eugene V. Koonin, National Center for Biotechnology Information, National Library of Medicine, National Institutes of Health, Bethesda, MD 20894, USA

This is a short but interesting story. The authors convincingly show that the DSB repair factor Cernunnos-XLF undergoes positive selection/adaptation (5 replacements against 0 synonymous substitutions in the human line since the divergence from the common ancestor with chimpanzee). It is proposed that XLF prevents neuronal apoptosis by improving the efficiency of DSB repair and thus provides for an increase in the number of brain neurons. To me, this speculation runs a little thin, i.e., I do not think that it is the only interpretation compatible with the data. I think it is impossible to rule out the possibility that XLF has another, still uncharacterized, perhaps, brain-specific function that is modified by the replacements in the human lineage; it is well known that protein moonlighting is common. However, nothing contradicts the authors' hypothesis either, and it could be argued that, for the moment, this is the most parsimonious explanation of the data.

**Author response**: *We agree with reviewers that the role of Cernunnos-XLF in human brain expansion is speculative. In the last paragraph of the Discussion we wrote that: "While association of Cernunnos-XLF selection with increased brain size is attractive in the context of simultaneous adaptation of several brain size determinants, there are also other possible explanations." Two such explanations are mentioned in the same paragraph. In the Abstract, we clearly stated that Cernunnos-XLF "possibly played a role" in human brain expansion. So far little is known about its function. However, the involvement of other positively selected candidate gene microcephalin/MCPH1/BRIT1 in DNA damage response (Lin et al, 2005) indicates that efficient DNA repair can be crucial in early brain development. In this context, the proposed selection on the repair factor Cernunnos-XLF is speculative, but in line with some current proposals on the mechanisms of human brain expansion*.

I believe that the paper would benefit from a somewhat more complete presentation of the evolutionary genomics of XLF. At least, I think it makes a lot of sense to point out that XLF is conserved in all animals and most fungi, as well as Dictyostelium, although the fungal and slime mold orthologs have a distinct domain architecture.

**Author response**: *Very recently a new paper appeared in JBC (Callebaut et al. 2006 J Biol Chem. 2006 Mar 29; ref 6) that partially addresses this question. In turn we modified the Introduction to "The Cernunnos-XLF protein shows similarity to XRCC4 *[5]*and is homologous to the Nej1 NHEJ factor from yeast *[6]. *The locus seems to be present in all animals, most fungi, but not in plants."*

Further, it is not quite correct to state that XLF has no sequence similarity to XRCC4; such similarity is detectable, as correctly pointed out by Ahnesorg et al, even if it is hard to demonstrate statistical significance.

**Author response**: *We agree and the sentence was corrected (see the previous point)*.
